# Telemedicine in the sky: prospects and challenges for the aviation sector in Sub Saharan Africa

**DOI:** 10.3389/fdgth.2026.1865607

**Published:** 2026-07-23

**Authors:** Sharon R. T. Chilunjika, Patrick Ngulube

**Affiliations:** School of Interdisciplinary Research and Graduate Studies, University of South Africa, Pretoria, South Africa

**Keywords:** challenges, commercial aviation, in-flight medical emergencies, prospects, telemedicine

## Abstract

The rapid growth of commercial aviation in Sub-Saharan Africa (SSA), combined with an increasing prevalence of chronic disease among travelers and limited in-flight medical resources, has elevated in-flight medical emergencies as a significant public health concern. Telemedicine presents a transformative opportunity to enhance aeromedical care. However, its adoption within SSA's aviation sector remains critically underdeveloped and underexplored in the literature. This integrative review examines the prospects and challenges of integrating telemedicine into SSA's aviation sector and generates context-specific recommendations to inform policy and practice. We conducted a comprehensive literature search across Scopus, Web of Science, PubMed, African Journals Online and Google Scholar, supplemented by grey literature. Thirty-five sources were synthesized across four analytical domains, with Rogers’ Diffusion of Innovations (DOI) theory providing the theoretical framework for the analysis. Six prospect domains were identified: real-time teleconsultations; multimodal biometric data transmission; continuity-of-care; psychosocial benefits for passengers and cabin crew; financial and operational/brand gains all aligning with the DOI attribute of relative advantage. However, these prospects are constrained by persistent challenges including unreliable satellite connectivity, ageing aircraft fleets, fragmented regulatory frameworks, cybersecurity vulnerabilities, financial barriers and inadequate cabin crew training. Recommendations are directed at three stakeholders namely, airline operators, aviation regulators and health policymakers and are anchored in regional realities. The review concludes that telemedicine holds transformative potential for SSA's expanding aviation sector, yet its realization demands harmonized regulatory action, targeted investment and capacity building tailored to the regions’ realities. Robust primary research within SSA's aviation health context is urgently needed to move beyond inferential evidence.

## 1 Introduction

Over the past two decades, the rapid growth of commercial aviation has transformed air travel from a privilege accessible to few into an indispensable mode of transportation for millions of people globally (1, 2). Concurrent with this expansion, technological advancements have catalyzed significant innovation in aeromedical care, opening novel frontiers for the delivery of emergency medical services in-flight (3, 4). As passenger numbers continue to rise, particularly within emerging markets, the incidence of in-flight medical emergencies has correspondingly increased (2). Statistical estimates indicate that such emergencies occur at a rate of approximately 10 to 50 cases per million passengers, a figure projected to grow in tandem with sustained expansion in global air travel (5). The confluence of increasing passenger numbers, aging traveler demographics and the rising prevalence of chronic medical conditions has elevated in-flight medical emergencies from an occasional inconvenience to a significant public health concern of global proportions (6).

The existing body of literature has systematically characterized the nature and distribution of medical incidents occurring during commercial flights (7, 8). Respiratory distress, syncope and near-syncope, gastrointestinal complaints and cardiovascular events are the most frequently reported categories of in-flight medical emergencies across peer-reviewed studies (7–9). In recent years, airlines have increasingly taken the role of managing these events in-flight, marking a significant departure from traditional, facility-based models of healthcare delivery (4, 10). Nonetheless, the infrastructure supporting in-flight health care delivery remains critically limited (1, 11, 12). The absence of a physician on commercial flights has been reported in approximately 41.1% of cases, largely owing to the logistical and financial unfeasibility of assigning dedicated health care across millions of flights operating annually (12). Furthermore, commercial aircrafts are typically equipped with only three categories of medical equipment: a basic first-aid kit, an emergency medical kit containing limited formulas of medications and diagnostic tools, and in some cases, an automated external defibrillator (1). These resources, while crucial, remain inadequate to address the increasing complexity and frequency of in-flight medical events (2, 6). As aircrafts travel longer routes and transport medically diverse passenger populations, the limitations of onboard medical kits and first-aid training among cabin crew have become increasingly important (11). To mitigate these constraints, ground-based medical support has emerged as an important adjunct in the provision of in-flight medical care (13).

Telemedicine is defined by (14) as the delivery of healthcare services using information and communication technologies (ICTs) where distance is a critical factor, encompassing services ranging from basic telephone consultations to advanced real-time consultations, has progressively gained prominence within aviation operations. In previous decades, telephone medicine, a foundational form of telemedicine, became established in commercial aviation, enabling cabin crew and volunteer medical professions- licensed clinicians travelling as passengers who self-identify during emergencies to receive real-time guidance from ground-based physicians (7, 15). More recently, the integration of advanced satellite communication systems into commercial aircraft has significantly expanded the technological potential for advanced telemedicine applications (16). These systems now support the transmission of physiological data, live video feeds and communication between in-flight and ground-based medical teams (3). These advancements are increasingly being credited for enabling more accurate diagnostic assessment and timely clinical decision-making (4, 17). Note that these technological capabilities have laid the groundwork for a paradigm shift from reactive telephone support to proactive, data-driven telemedicine within aviation operations. These developments mirror broader transformations in global healthcare provision, where telemedicine has increasingly become a cornerstone of remote patient management and emergency response (18).

Despite these global advances, the adoption of telemedicine within aviation operations across Sub- Saharan Africa (SSA) remains critically limited in both research and practice. The extant literature on in-flight telemedicine is mainly concentrated in high income economies, where robust telecommunication infrastructure, mature regulatory frameworks and well-resourced healthcare systems provide fertile grounds for technological integration (4, 18). On the other hand, airlines operating within SSA face distinctive systemic and operational challenges seldom addressed in aviation medicine research. These include limited access to real-time ground-based medical consultation services, regulatory fragmentation across national borders, and persistent gaps in satellite and radio communications along transcontinental and remote flight corridors (19–21). Furthermore, the intersection of telemedicine and aviation medicine with the broader landscape of developing health systems, particularly in SSA, has received limited scholarly attention. Research on telemedicine in SSA has primarily focused on terrestrial healthcare delivery (22, 23) emphasizing primary and community care rather than highly specialized, time-critical environments of in-flight medicine. This represents a significant gap in the literature, particularly given the rapid expansion of the aviation market across SSA and the disproportionate burden of both non-communicable and communicable diseases among its traveling population. Addressing this knowledge gap is crucial to align aviation healthcare preparedness with the region's growing air transport demands, and to contribute meaningfully to the global discourse on aviation medicine.

Due to the paucity of literature directly addressing the integration of telemedicine within SSA's aviation sector, this review addresses a demonstrable gap in the literature by exploring the prospects and challenges of telemedicine adoption in SSA's aviation sector, generating a foundational framework to anchor future empirical inquiry and policy development. In so doing it draws on three convergent bodies of adjacent evidence: global studies documenting the benefits and challenges of telemedicine integration in the aviation sector, aviation medicine practices in developing and resource-constrained contexts and telemedicine adoption within SSA's broader healthcare systems. Each domain contributes a distinct analytical layer that, taken together, enables an inferential but structured account of the SSA aviation telemedicine landscape. The contribution of this review is therefore to perform the necessary prior intellectual work of mapping the landscape and identifying the structural and contextual variables most likely to shape adoption dynamics. Specifically, the review seeks to answer the following questions:

What are the prospects of telemedicine deployment in the SSA aviation sector?
What are the primary challenges that can impede the successful implementation of telemedicine in the SSA aviation sector?

## 2 Literature review and theoretical framework

### 2.1 An overview of telemedicine in the aviation sector

Telemedicine in aviation refers to the integration of remote health services and digital technologies into the aviation sector, to support and oversee the health needs of passengers and crew members during flights (3, 16). It involves the use of telecommunication technologies including secure messaging, video conferencing and real-time data transmission to allow direct communication between aviation crew and clinicians on the ground (18). The integration of telemedicine in aviation represents a significant advancement in passenger safety and emergency response (3). With telemedicine technologies on board, flight crews operating at technologically mature carriers can share live videos feeds, vital signs and other relevant patient data with ground-based professionals in real time (4, 16). Within SSA, this capability is currently most achievable among leading carriers operating modern fleets, such as Ethiopian Airlines and Kenya Airways. This enables expert visual assessment, guided diagnosis and accurate treatment recommendations that improve the quality of in-flight medical care (24–26). Additionally, it maximises medical resource utilization and reduces disruptions caused by medical emergencies during flights (4). Emirates and KLM Royal Dutch Airlines are examples of airlines using telemedicine on their long-haul flights (16). These services have been credited for resolving in-flight emergencies without the need for diversion (16).

Beyond emergency response, telemedicine in aviation encompasses pre-flight screening and pre-boarding consultations (16, 18). This facilitates proactive health assessment and risk mitigation prior to travel (16, 18). Note that these services enable the early identification and management of potential medical emergencies, ensuring that passengers are clinically fit for travel. Additionally, reducing the likelihood of in-flight medical events (18). Airlines such as Lufthansa for example, provide pre-flight telemedicine consultations that allow passengers to seek medical advice and obtain clearance prior to their trip (16). Such practices contribute to a more seamless travel experience while enhancing operational preparedness (27). Where passengers disclose pre-existing medical conditions in advance (16), states that airlines are better positioned to design individualized care plans and ensure that appropriate medical resources are available onboard (16). further alludes that this preparedness enables effective coordination of contingency measures, yielding dual benefits of enhanced patient safety and operational efficiency.

In the era of the Fourth Industrial Revolution (4IR), the adoption of telemedicine in aviation has become increasingly imperative for both developed and developing countries. Evidence indicates that telemedicine has evolved from a supplementary tool to an integral component of modern healthcare delivery, driven by advances in digital connectivity, artificial intelligence and real-time data exchange (26, 27). Studies by (23) and (28) demonstrate that telemedicine improves access to care, enhances clinical outcomes and enhances timely interventions, particularly in resource constrained or geographically isolated settings. In aviation, where medical resources are inherently limited, these capabilities are important for ensuring continuity and quality of care. Moreover, rising global passenger volumes and increasing prevalence of chronic conditions further necessitate scalable, technology-enabled health solutions (27). This positions telemedicine as a crucial innovation for resilient and future ready aviation systems (4).

### 2.2 Diffusion of innovations theory (DOI)

This review adopted Rogers' Diffusion of Innovations (DOI) theory (29). The theory provides a robust theoretical lens for examining how and why airline operators, regulatory stakeholders and healthcare professionals adopt or resist telemedicine within the aviation sector in SSA. At its core, DOI posits that the rate of adoption of any innovation is shaped by five perceived attributes: Relative Advantage, Compatibility, Complexity, Trialability and Observability (29). These attributes are particularly salient in the context of aviation telemedicine in SSA, where the interplay of infrastructural barriers, technological readiness and medical exigency shape adoption dynamics in ways that are both complex and context specific. DOI further conceptualizes adoption as a social process mediated by communication channels, time and the social systems within which organizations operate, enabling a systemic rather than purely individual-level analysis of adoption patterns across SSA's heterogeneous aviation sector.

Relative Advantage, defined as the degree to which an innovation is perceived as better than the idea, practice or technology it supersedes (29), directly maps onto the prospects of telemedicine in SSA's aviation sector. In the context of in-flight telemedicine, Relative Advantage is demonstrated through improved clinical outcomes and operational efficiency. Evidence from aviation health studies indicates that real-time video consultations and biometric data transmission can expedite medical decision-making and reduce flight diversion (4). For airlines across SSA, where passenger volumes are increasing despite limited in-flight medical expertise, such capabilities are particularly valuable. Telemedicine has the potential to reduce costly diversions, enhance passenger safety and support airline compliance with safety-related health regulations. Evidence from global aviation studies supports this, showing that telemedicine interventions can reduce emergency response times by up to 40 percent compared with conventional radio communication (18). These findings are further reinforced by evidence from developing healthcare systems, where perceived clinical utility has consistently emerged as a key determinant of telemedicine adoption (22). This reinforces the centrality of Relative Advantage in shaping adoption trajectories within the SSA aviation sector.

Complexity, defined as the degree to which an innovation is perceived as relatively difficult to understand and use (29), addresses how potential adopters assess the effort required to operate telemedicine systems effectively. In SSA's aviation context, Complexity is shaped by factors such as unreliable satellite connectivity at cruising altitude, inconsistent broadband penetration and significant disparities in digital health literacy among airline medical staff. DOI posits that when potential adopters perceive an innovation as technically demanding, its rate of adoption will be constrained regardless of its value (29). Understanding Complexity thus helps to explain persistent adoption gaps in aviation telemedicine, where technological optimism is tempered by structural limitations and human capital constraints. Importantly, attending to the Complexity attribute does not only illuminate implementation barriers, but also identifies actionable areas for capacity building and user-centred design to advance telemedicine adoption in SSA's aviation sector.

Beyond Relative Advantage and Complexity, DOI identifies three further innovation attributes that shape adoption rates. Compatibility refers to the degree to which an innovation is consistent with existing values, past experiences and the needs of potential adopters (29). In the SSA aviation context, telemedicine must be compatible with prevailing operational workflows, cultural attitudes toward digital health and existing aviation regulatory frameworks. Trialability denotes the degree to which an innovation may be experimented with on a limited basis, while Observability captures the extent to which the results of adoption are visible to others in the social system. These attributes are particularly relevant to the SSA context, where limited prior exposure to aviation telemedicine constrains the diffusion process. The absence of harmonized policy frameworks and regulatory fragmentation across African Civil Aviation Commission (AFCAC) member states (21) further undermines compatibility, creating institutional uncertainty that hampers adoption. DOI additionally distinguishes adopter categories ranging from innovators and early adopters through to the early majority, late majority and laggards, a typology that maps directly onto the heterogeneity of SSA's aviation sector. Leading carriers such as Ethiopian Airlines and Kenya Airways may be characterized as early adopters, while smaller regional carriers operate within late majority or laggard categories where structural constraints foreclose timely adoption. These adopter dynamics, mediated by communication channels and the structure of the aviation social system, mean that telemedicine diffusion in SSA cannot be reduced to individual organizational decisions but must account for systemic and policy level enablers and constraints.

In sum, Rogers' DOI provides a theoretically coherent and empirically grounded framework for exploring the prospects and challenges of telemedicine adoption in SSA's aviation sector. By aligning the region's infrastructural, regulatory, human capital and technological realities with the theory's five innovation attributes and adopter category typology, this review advances a nuanced understanding of adoption dynamics that is both globally informed and locally responsive. The theory positions aviation telemedicine not simply as a technological intervention but as a sociotechnical innovation whose diffusion is shaped by perceived attributes, communication channels, time and the structure of the adoption social system. Given the unique sociotechnical landscape of SSA, DOI provides not only a lens for understanding current adoption gaps but also a framework that bridges theoretical insight with actionable implications for policy, practice and future research in SSA's aviation sector.

Notwithstanding its utility, DOI carries limitations worth acknowledging. The theory was originally developed to explain the adoption of agricultural innovations and its application to complex sociotechnical systems in healthcare and aviation requires careful contextual adaptation (29). Its five innovation attributes, while analytically useful, offer limited explanatory reach over organizational power dynamics, political economy constraints and the structural determinants of technology access that are particularly salient in SSA. Another limitation of DOI in this context is that its diffusion logic assumes adopters possess sufficient agency, financial capacity and infrastructure to act on perceived innovation attributes. However, in SSA's aviation sector, these preconditions are largely absent for most carriers. This review therefore applies DOI as a diagnostic lens to map where structural preconditions for adoption are met or absent across SSA's heterogeneous aviation sector, rather than as a prescriptive model predicting adoption behavior.

While the Technology Acceptance Model (TAM)'s perceived ease of use closely mirrors DOI's Complexity construct, and the Unified Theory of Acceptance and Use of Technology (UTAUT) offers complementary institutional perspectives, both frameworks prioritize individual-level acceptance over system-level diffusion dynamics. DOI was selected specifically because this review examines adoption across a heterogenous multi- organizational aviation sector, where adopter category typology, communication channels and social system structure provide greater analytical utility than individual behavioral intention models. Furthermore, DOI was selected for its capacity to account for systemic and social dimensions of innovation diffusion, its established utility in health technology adoption studies (30), and its direct mapping onto the prospect-challenge structure of this review, particularly the heterogeneity of adoption readiness across SSA's aviation sector.

## 3 Methods

This review adopted an integrative review methodology, following the guidelines established by (31). The integrative review methodology was selected for its capacity to synthesize diverse evidence sources within a structured and transparent process. This approach is particularly suited to the present study given the paucity of primary research directly investigating telemedicine implementation in SSA's aviation sector, necessitating synthesis across adjacent disciplinary fields.

### 3.1 Review design rationale

Unlike systematic reviews that restrict inclusion to controlled studies, integrative reviews accommodate heterogeneous methodologies, thus enabling a comprehensive appraisal of an emerging and underexplored topic (31). This integrative design enables this review to draw on multiple study designs and grey literature, all of which are necessary to address the multi-dimensional prospects and challenges of telemedicine deployment in SSA aviation. This review was not registered prospectively given its integrative rather than systematic nature. Nonetheless, the review protocol, including search strategy, eligibility criteria and data extraction procedures, was defined prior to database searching to minimize *post hoc* decision-making. The paucity of primary literature directly addressing telemedicine implementation within SSA's aviation sector necessitates an inferential synthesis strategy, a methodologically legitimate and well-precedented approach in nascent fields where direct evidence has yet to accumulate (32). Rather than treating this evidentiary gap as a limitation that impedes scholarly contribution, this review adopts it as the defining rationale for an integrative approach that traverses adjacent disciplinary domains. Evidence is drawn from three convergent bodies of adjacent evidence: global studies documenting the benefits and challenges of telemedicine integration in the aviation sector, aviation medicine practices in developing and resource-constrained contexts and telemedicine adoption within SSA's broader healthcare systems. Each domain contributes a distinct analytical layer that, taken together, enables an inferential but structured account of the SSA aviation telemedicine landscape. The contribution of this review is set the foundation that can anchor and direct future research in this underexplored area.

### 3.2 Literature search

We conducted a comprehensive literature search across multiple databases, including Scopus, Web of Science, PubMed, African Journals Online (AJOL) and Google Scholar between December 2025 and February 2026. Boolean operators “AND” and “OR” were used to combine the search terms. Keywords including telemedicine, telemedicine, digital health, e-health, remote consultation, teleconsultation, Sub-Saharan Africa, Africa, developing countries, aviation medicine, aerospace medicine, aeromedical, cabin crew, air passengers and in-flight emergencies were used in various combinations to retrieve relevant literature. A preliminary database search revealed limited peer-reviewed publications addressing telemedicine integration in SSA's aviation sector. Therefore, the search strategy deliberately incorporated sources from adjacent disciplinary domains, grey literature and institutional repositories to construct a sufficient evidentiary base for inferential synthesis. Sources included technical reports, policy documents and working papers from International Civil Aviation Organization (ICAO) and the WHO. Supplementary search strategies included manual reference list screening of key articles and citation tracking of seminal works on telemedicine integration in the aviation sector.

**Figure 1:**
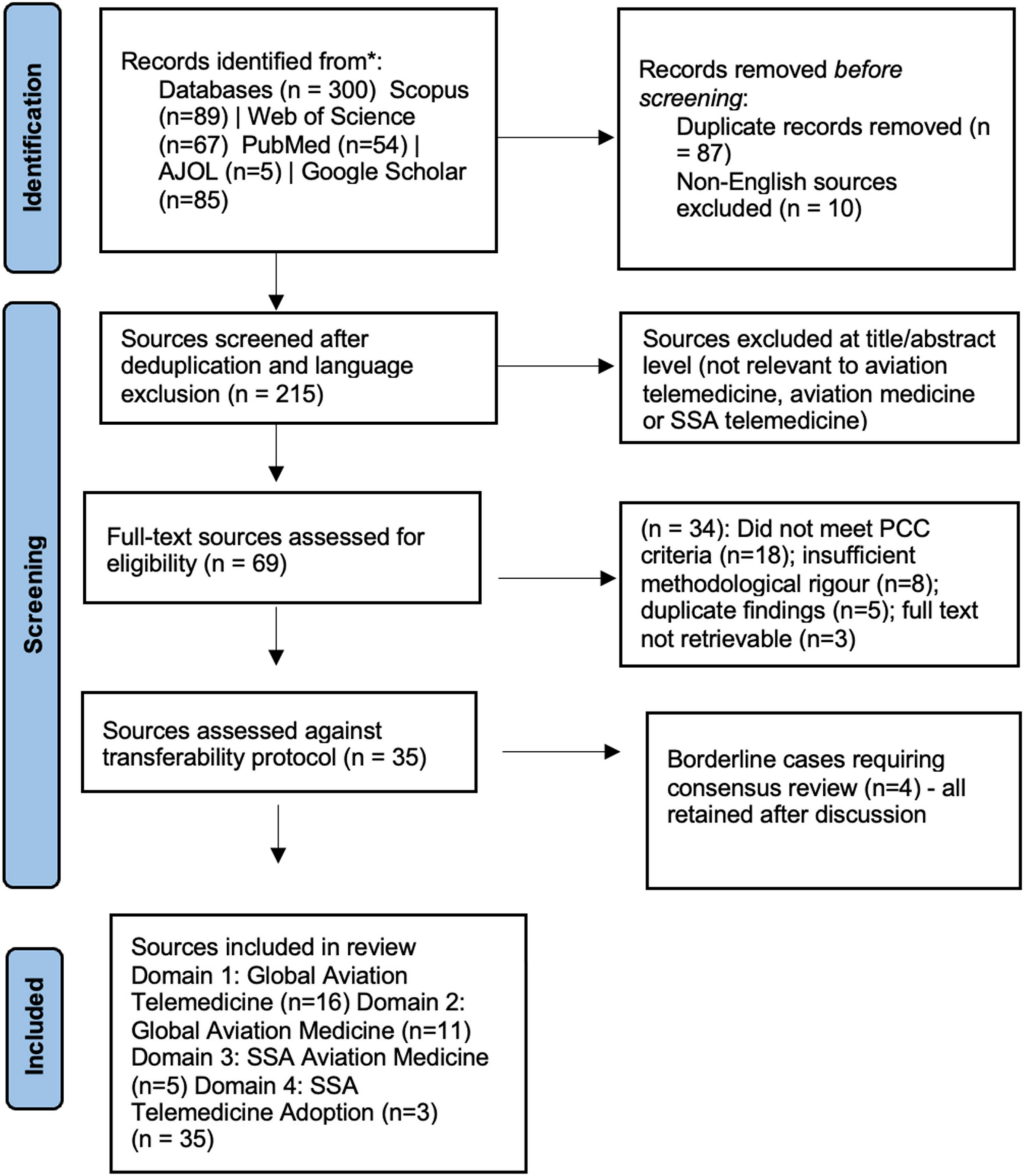
PRISMA flow diagram.

The search and selection process is summarized in Figure 1. Database searching yielded 300 records, supplemented by 12 additional sources identified through grey literature and manual referencing screening, yielding 312 records. Following removal of 87 duplicate records and exclusion of 10 non-English sources, 215 records were screened at title and abstract level, of which 146 were excluded as not relevant to aviation telemedicine, aviation medicine or SSA telemedicine. The remaining Sixty-nine full-texts sources were assessed for eligibility, and 34 were for the following reasons: did not meet PCC eligibility criteria (*n* = 18), insufficient methodological rigor (*n* = 8), duplicate findings already represented (*n* = 5) and full text not retrievable (*n* = 3). All remaining sources were evaluated against a four-criteria transferability protocol, with four borderline cases resolved through consensus discussion. Thirty-five sources were ultimately included and organized across four analytical domains for syntheses.

### 3.3 Inclusion and exclusion criteria

To ensure rigor and reproducibility, eligibility criteria were defined using a Population, Concept, Context (PCC) framework. Given the acknowledged paucity of peer-reviewed literature addressing telemedicine in SSA's aviation sector, the PCC framework was intentionally designed to accommodate adjacent disciplinary evidence. Table 1 summarizes the inclusion and exclusion criteria applied during the screening process*.* No publication date restriction was applied to the search strategy. Given the limited volume of literature directly addressing telemedicine in SSA's aviation sector, imposing date cutoff risked excluding foundational evidence in an underexplored field. While older sources such (35, 37) predate modern satellite connectivity advances, they were retained not as representations of current technological capacity but for their enduring relevance to medico-legal, regulatory and organizational dimensions of aviation telemedicine that remain unresolved.

**Table 1:** Inclusion and exclusion criteria.

Criterion	Inclusion	Exclusion
Population	Studies involving any of the following: commercial airline passengers; cabin crew and aviation medical officers; ground-based medical support personnel; healthcare providers delivering remote teleconsultations services to aviation settings; airline operators and regulatory authorities involved in in-flight health service governance.	Studies focused exclusively on hospital inpatients, non-aviation emergency services, or general community health populations with no relevance to in-flight or aeromedical contexts.
Concept	Studies addressing one or more of the following: telemedicine or telemedicine adoption, implementation or integration in aviation settings, in-flight medical emergency management using digital health technologies, prospects benefits or clinical outcomes of telemedicine adoption in aviation or LMIC emergency care contexts, telemedicine adoption in global aviation contexts	Studies on general healthcare delivery with no aviation or aeromedical relevance, opinion pieces, editorials and blog posts lacking empirical or institutional evidence base
Context	Studies conducted in SSA; global aviation medicine studies, LMIC and developing-country telemedicine studies with transferable implications for SSA (assessed using the transferability protocol discussed in the subsequent section)	Studies exclusively focused on high income settings with no transferable implications for SSA, assessed against the Transferability protocol discussed in the subsequent section.
Language	Articles and reports published in English	Publications in languages other than English

### 3.4 Transferability assessment protocol

The Geographic scope exclusion required a judgement about whether evidence generated in high-income settings carried transferable implications for SSA. To make this determination reproducible and minimize reviewer subjectivity, transferability was assessed against four pre-specified criteria. A study was deemed to have transferable implications for SSA's aviation sector if it met one or more of the following: (1) the study setting shared at least one structural analogue with SSA aviation contexts, defined as limited specialist workforce availability, resource-constrained healthcare infrastructure or fragmented regulatory environments; (2) the study explicitly situated its findings within LMIC or developing country contexts, or discussed scalability constraints applicable to under-resourced health systems; (3) the barrier, intervention, or outcome described was infrastructure-dependent in a manner directly applicable to SSA's satellite connectivity limitations, ageing fleet profiles, or coverage gaps along intra-African flight corridors; or (4) the study addressed regulatory, financial, or organizational challenges independently documented in SSA aviation literature, for example (19–21).

To enhance the transparency and reproducibility of the transferability assessment, both authors independently evaluated all 35 sources against the four pre- specified criteria. A decision log was maintained throughout to record the rationale for each inclusion or exclusion judgment. Of the 35 sources screened, 31 were assessed as transferable without requiring discussion. The remaining 4 borderline cases, specifically (7, 11, 37, 40), were predominantly high-income or jurisdiction specific studies from Domains 1 and 2 (In Supplementary Table 1) that required structured consensus review between both authors. All were ultimately retained following discussion, as each addressed infrastructure, regulatory or organizational dimensions independently documented in SSA aviation literature.

### 3.5 Data extraction

In total 35 sources were included in this review. Using a structured extraction sheet, the following data were gathered from each source, authors, publication date, study design, geographic setting and key findings (see Supplementary Table 1). The 35 included sources were organized across four analytical domains reflecting the review's inferential synthesis strategy. Domain 1, comprising global aviation telemedicine studies contributes the largest body of evidence with 16 sources. Domain 2, covering global aviation medicine studies with specific focus on in-flight medical emergency epidemiology and clinical management, contributed 11 sources. Domain 3 consisting of aviation medicine studies in developing and resource-constrained contexts within SSA contributed 5 sources. Domain 4, drawing on telemedicine adoption studies within SSA's broader healthcare systems contributed 3 sources. Following data extraction, thematic synthesis was conducted through an iterative, interpretive process informed by DOI theory. The primary author conducted the initial coding with regular verification and collaborative discussion with the second author to maintain analytical rigor and minimize interpretive bias.

## 4 Results

### 4.1 Prospects of telemedicine adoption in SSA's aviation sector (RQ1)

The review explored the prospects of telemedicine adoption in SSA's aviation sector, addressing research question 1. The findings are summarized in Table 2.

**Table 2:** Prospects of telemedicine adoption in aviation.

Category	Benefit	Authors	DOI attribute (s)
Clinical and diagnostic	Real time consultation between cabin crew and ground clinicians via satellite and air-to ground links. Transmission of patient data (e.g oxygen saturation, live video, ECG for remote decision making. Physician-assisted oversight supports safe continuation of most flights. Faster, more accurate diagnosis through multimodal biometric data Pre-flight passenger data gathering	(4, 16, 18, 24)	Relative advantage;
Continuity of care	Early coordination with ground healthcare facilities. Creation of auditable in-flight clinical records for handover	(4, 7, 15, 36)	Trialability; Relative advantage
Psychosocial (passengers)	Awareness of telemedicine services enhances perceived safety and satisfaction. Reassurance for passengers with chronic illness or travel-related anxiety.	(16, 18)	Relative advantage; Observability
Psychosocial (cabin crew)	Greater confidence and lower distress among crew using telemedicine support. Real-time clinician guidance reduces fear of medical error and improves perceived competence.	(8, 25, 26, 36)	Relative advantage; Observability
Financial	Remote triage reduces unnecessary route diversions. Reduction in fuel, crew overtime, airport charges and rebooking costs.	(16, 18)	Relative advantage
Operational (Brand & trust)	Adoption of telemedicine systems signals strong commitment to health and safety. Documented remote consultations support medico-legal accountability and quality assurance	(10, 16, 27)	Relative advantage; Observability

### 4.2 Challenges to telemedicine adoption in SSA's aviation sector (RQ2)

The adoption of telemedicine within SSA's aviation sector faces several challenges which can be broadly categorized into technological, legal, financial and organizational factors. These results are tabulated in Table 3.

**Table 3:** Challenges to telemedicine adoption in aviation.

Category	Challenge/Barrier	Source
Technological	Limited broadband and unstable connectivity Ageing aircraft and incompatible onboard systems.	(3, 4, 17, 37)
Legal and regulatory	Absence of clear cross-border telemedicine regulatory framework. Unclear medico-legal liability for telemedicine decisions. Inadequate data protection and privacy legislation. Weak cybersecurity for in-flight health data	(18, 35, 37, 40)
Financial	High capital investment required for the procurement and installation of onboard telemedicine equipment, satellite communication systems and compatible ground-based infrastructure. Limited public or institutional funding mechanisms to subsidise telemedicine adoption in the aviation sector.	(18, 19, 39)
Organizational	Limited awareness and prioritization of telemedicine among airline leadership. Inadequate training and capacity among cabin crew. Shortage of aviation medical officers and 24/7 ground physician support.	(11, 15, 18, 20)

### 4.3 Discussion

The uneven distribution of evidence across the four analytical domains see (Supplementary Table 1) constitutes a key finding of this review. In particular, Domains 3 and 4, representing SSA specific aviation and telemedicine literature collectively account for only eight sources. This stark imbalance provides direct empirical support for the review's primary claim that regionally grounded evidence in on telemedicine adoption in aviation remains critically underdeveloped. Accordingly, the greater reliance on global literature is not indicative of selection bias but rather reflects an analytically necessary response to this documented gap. This pattern highlights the current limitations of the regional evidence base and underscores an urgent need for more SSA-focused primary research.

#### 4.3.1 Prospects of telemedicine in SSA's aviation sector (RQ1)

Before discussing the findings, it is important to note, that SSA is not a homogeneous entity. The region encompasses 46 countries with markedly different aviation profiles, regulatory maturity, health system capacities and digital infrastructure. Frontier aviation markets including Ethiopia, Kenya and South Africa, home to Ethiopian Airlines, Kenya Airways and Airlink, operate at a fundamentally different level of technological readiness than carriers in fragile or least developed country contexts where domestic aviation infrastructure remains minimal (21). Consequently, the prospects discussed below should not be read as uniformly attainable across SSA, but rather as differentially realizable depending on each carrier's fleet profile, connectivity infrastructure and institutional capacity. Nonetheless, the synthesized evidence suggests that SSA's aviation sector stands to benefit from telemedicine adoption, provided the enabling conditions are progressively addressed.

The clinical and diagnostic benefits identified in Table 2, including real-time teleconsultation, multimodal transmission of patient data, pre-flight patient data collection and clinician-assisted decision support, align with the DOI attribute of Relative Advantage as they demonstrate telemedicine 's capacity to enhance clinical performance in the aviation environment. This is substantiated at the global aviation level by a well-established body of evidence (4) and (16) collectively document that real-time video consultations and biometric data transmission expedite in-flight clinical decision-making and reduce the frequency of unnecessary flight diversions (16). further notes that airlines including Emirates and KLM Royal Dutch Airlines have deployed advanced telemedicine systems on long-haul fleets, with these services credited for resolving cardiovascular and other emergencies without diversion. Critically, these global examples also illustrate the DOI attribute of Trialability. Emirates and KLM's deployments were themselves incremental, beginning with ground-based teleconsultation agreements before progressing to integrated onboard systems. This demonstrates that aviation telemedicine is not an all-or-nothing investment but a scalable innovation flexible to phased experimentation (16). For SSA carriers, this is a strategically significant finding. Airlines such as Ethiopian Airlines and Kenya Airways, which already operate satellite-equipped fleets, are positioned to trial ground-based teleconsultation services through service agreements with established providers such as MedAire's Aviation MedLink, without requiring immediate onboard hardware investment. This low-barrier entry point raises the Trialability of telemedicine for SSA's more advanced carriers, enabling early adoption experience to accumulate before full-scale system integration is pursued.

Underpinning these clinical benefits is the interaction architecture through which telemedicine is delivered in-flight. Consultations predominantly follow a triadic model, which is the most operationally realistic structure given that satellite communications are routed through crew and liability frameworks place medical management responsibility on airline personnel rather than the patient (18, 38). Within the DOI, this triadic structure raises the Complexity of telemedicine deployment, as it demands coordinated communication competencies across multiple actors (8, 25, 26). Decision support systems, however, partially mitigate this Complexity by structuring crew-clinician interaction and reducing perceived error risk (8). Emerging AI- assisted diagnostic tools further hold potential to improve real-time clinical decision-making and reduce dependence on ground physician availability (12, 13, 15), strengthening the Relative Advantage of telemedicine. Nonetheless, the scope and quality of care deliverable in-flight particularly in SSA's developing context remains constrained by onboard equipment limitations (11), reinforcing telemedicine as a decision -support system, rather than a substitute for comprehensive care.

Additionally, Table 2 highlights pre-flight passenger data collection as a clinical advancement that shifts telemedicine from a reactive emergency tool to a proactive system of healthcare. Studies by (18) and (16) show that pre-flight telemedicine consultations enable passengers with pre-existing conditions to disclose their health status in advance, allowing airlines to allocate appropriate medical resources and develop individualized care and contingency plans. This benefit is of significant value in SSA, given the disproportionate burden of non-communicable diseases including diabetes, hypertension and cardiovascular disease and communicable diseases among SSA's travelling population (33). specifically document the elevated risk of in-flight complications among SSA passengers with conditions such as sickle cell disease, highlighting the clinical necessity of pre-flight risk stratification tailored to the region's disease profile. Similarly (34), note that the epidemiology of in-flight medical events on African airline carriers reflects the specific disease burden of the region's passenger population, reinforcing the value of context-specific screening over universal protocols derived from high-income settings.

Pre-flight and in-flight telemedicine, however, differ significantly in both technical infrastructure and clinical objectives, a distinction with direct implications for adoption prospects and challenges in SSA. Pre-flight telemedicine purses proactive clinical objectives including passenger fitness assessment, chronic disease disclosure and individualized care planning within in a stable ground-based environment requiring only standard internet connectivity and existing health system infrastructure. This thus, lowers technical Complexity and facilitates higher Compatibility with traditional workflows (16, 18). In-flight telemedicine, by contrast, pursues reactive emergency objectives such as real-time triage, diagnostic support and diversion-avoidance decision making under time critical constraints demanding satellite connectivity, onboard hardware integration, significantly raising Complexity and reducing Trialability (4, 17). For SSA carriers, this distinction positions significant, pre-flight services as a more immediately achievable adoption entry point (16, 21).

The continuity-of-care benefits documented in Table 2, particularly the generation of auditable in-flight clinical records and early coordination with ground healthcare facilities, reinforce both the Relative Advantage and Trialability of aviation telemedicine in the SSA context. At the global level (35) and (15) establish that structured telemedicine -enabled documentation supports post-event review and medico-legal accountability, while (36) and (16) demonstrate that early ground coordination improves post-landing patient outcomes by reducing delays in time-critical interventions. From a Trialability perspective, continuity-of-care functions are particularly flexible to limited-scale experimentation. They can be initiated through existing communication infrastructure, including radio and basic satellite links already operational on many SSA aircraft, without requiring full telemedicine system installation. Additionally, structured clinical documentation protocols and ground coordination procedures can be piloted on selected high-frequency routes, generating observable outcome data that builds institutional confidence and informs iterative system refinement before wider rollout. This incremental approach is consistent with DOI's proposition that innovations with high Trialability diffuse more rapidly, as the ability to test on a limited basis reduces perceived risk and lowers the threshold for adoption commitment (29). In the SSA context, where scepticism toward large-scale digital health investments is partly rooted in past implementation failures documented in terrestrial health systems (22, 23), the demonstrable pilotability of continuity-of-care functions represents a meaningful pathway for building institutional trust and adoption momentum across the region's aviation sector. This benefit is contingent, however, on the interoperability between in-flight and ground health systems, a persistent gap given the region's fragmented digital infrastructure. This means that Trialability gains are most readily achievable in digitally mature contexts such as South Africa, Rwanda, Kenya and Ethiopia, where ground health systems are better positioned to receive and act on telemedicine-generated data.

This differentiated achievability reflects a broader principle, that the clinical functionality and effectiveness of aviation telemedicine are not uniform but are materially determined by the infrastructural and operational context in which they are deployed. In high-income aviation contexts, stable satellite connectivity, modern fleet infrastructure and available ground physician support enable telemedicine to function at its full clinical potential, delivering real-time diagnostic support and continuity of care (4, 16). In SSA, however, these enabling conditions are unevenly distributed, constraining the clinical effectiveness of the same intervention. Unstable connectivity limits real-time data transmission quality, ageing fleets restrict onboard system integration and aviation medical officer shortages reduce ground-based clinical support availability. Within DOI, these contextual deficits simultaneously weaken the Relative Advantage, Compatibility and Trialability of telemedicine for the majority of SSA carriers. This means that clinical benefits documented in global literature may only be partially realized in the region's resource-constrained aviation environment (22, 23).

The psychosocial benefits including improved passenger confidence and reduced crew distress are also consistent with the DOI attribute of Relative Advantage and critically the attribute of Observability. Observability is particularly crucial here because psychosocial benefits are by nature visible. Passengers who witness crew engaging with ground clinicians via telemedicine systems, or who are informed that real-time medical support is available on board, receive a direct and legible signal of the airline's health and safety commitment (16) and (18) document that visible telemedicine capacity increases passenger perceptions of safety and airline trust, while (25) and (26) demonstrate that real-time clinical support measurably reduces cabin crew anxiety and perceived error risk during in-flight medical emergencies. In DOI terms, these outcomes are highly observable as they are experienced directly by passengers and crew during the in-flight emergencies. Furthermore, these benefits are communicated through word-of-mouth and airline reputation channels and are visible to regulators and competitors as markers of operational quality. This high Observability functions as a diffusion accelerant (29), as airlines that adopt telemedicine generate visible outcome signals that increase adoption willingness among peer carriers operating within the same social system.

Applied to the SSA context, the Observability of psychosocial benefits carries strategic significance for airline brand positioning in an increasingly competitive regional market. For carriers such as Ethiopian Airlines and Kenya Airways, whose route networks extend across both regional and intercontinental corridors, the visible deployment of telemedicine systems communicates a commitment to passenger welfare that differentiates them within a market where health and safety standards are increasingly scrutinized by international partners, insurers and regulatory bodies. Furthermore (10) and (27) note that documented remote consultations and telemedicine-supported emergency responses strengthen medico-legal accountability and contribute to quality assurance frameworks, outcomes whose visibility extends beyond individual passengers to institutional stakeholders. For smaller regional carriers, the Observability dynamic operates differently but remains relevant. As leading SSA carriers generate observable telemedicine outcomes, the diffusion logic of DOI predicts that early majority and late majority adopters within the region's aviation social system will face increasing normative pressure to follow, particularly where passenger expectations are shaped by exposure to telemedicine-equipped international carriers.

However, this review identifies a critical Observability gap that may limit the diffusion potential of psychosocial benefits within SSA's aviation sector specifically. Observability, as (29) conceptualizes it, is not merely about whether outcomes are visible in principle, but whether they are visible within the relevant social system. This means, observable to the specific adopters whose decisions shape diffusion trajectories in each context. The documented psychosocial and clinical outcomes of aviation telemedicine are mainly concentrated in high-income airline settings, with Emirates and KLM serving as the primary reference cases in literature (16).These outcomes are geographically and institutionally displaced from SSA's aviation decision-makers, regulators and cabin crew, meaning that Observability of adoption benefits operates at a distance rather than within the region's own social system. This displacement weakens its adoption-motivating effect. Where potential adopters cannot observe telemedicine outcomes among regional peers operating under comparable infrastructural and financial conditions, the persuasive force of global evidence is lessened, and adoption scepticism is reinforced. This is compounded by a dearth of documented passenger and crew perceptions of telemedicine within the SSA aviation context, a gap this review identified across all four analytical domains. Without locally observable evidence of psychosocial benefit, the Compatibility and Observability conditions that DOI identifies as central to adoption willingness (29), remain unmet for the majority of SSA carriers and their stakeholders. This evidence gap is a structural barrier to diffusion. It limits the peer visibility and regional learning, that DOI identifies as the primary social mechanism through which innovations move from early adopters to the early and late majority within a social system. Addressing it demands deliberate investment in primary research that generates and disseminates locally observable outcome data, a priority this review positions as foundational to advancing telemedicine diffusion across SSA's aviation sector.

#### 4.3.2 Challenges to telemedicine adoption in SSA's aviation sector (RQ2)

While the benefits identified in this review align with the DOI attribute of Relative Advantage, the challenges summarized in Table 3 directly illuminate the Complexity and Compatibility deficits that constrain diffusion. DOI posits that where an innovation is perceived as technically demanding or incompatible with existing values and infrastructure, its rate of adoption will be impeded regardless of its inherent advantages (29). The legal and regulatory challenges outlined in Table 3, mirror the global complexities surrounding the governance of telemedicine in aviation. Internationally, the absence of harmonized cross-border telemedicine frameworks and the persistence of unclear medico-legal liability for remote clinical decision-making continue to impede the broader integration of digital health systems into aviation practice (18, 37, 38). Telemedicine, as a technology predicated on seamless cross-jurisdictional data transmission and remote clinical decision-making, is structurally incompatible with the fragmented multi-jurisdictional governance environment that currently characterizes the global aviation landscape (37). Within the DOI framework, where an innovation cannot be reliably integrated into the prevailing governance architecture, the theory predicts that potential adopters will perceive it as incompatible with their operational environment (29), suppressing adoption willingness regardless of its clinical merits. This is particularly consequential for intra-African carriers whose routes cross multiple national jurisdictions, as medico-legal liability for remote consultations remains undefined at both the national and regional level (18, 38). Until regulatory frameworks across SSA member states are harmonized to formally accommodate telemedicine as a recognized mode of in-flight care, this Compatibility deficit will remain a systemic constraint on adoption.

The organizational challenges documented in Table 3 reflect systemic underinvestment in aviation health capacity that directly undermines Compatibility at the operational level. For telemedicine to align with existing workflows, cabin crew must be trained to manage new communication protocols and data-handling responsibilities, yet (11, 15, 20) consistently identify limited leadership prioritization, inadequate training and aviation medical officer shortages as recurrent barriers to implementation. In SSA, immediate operational and financial pressures further suppress leadership commitment to telemedicine investment, perpetuating training deficits that leave cabin crew without the clinical confidence or technical proficiency to deploy telemedicine systems during emergencies. Broader health workforce shortages across the region extend to aviation medicine, limiting the availability of qualified medical officers to provide continuous ground-based teleconsultation support, rendering telemedicine operationally incompatible with current workforce realities. These organizational deficiencies simultaneously heighten perceived Complexity, erode perceived Relative Advantage and deepen Compatibility deficits by undermining the institutional support, user competency and operational confidence on which successful adoption depends. Addressing them is therefore not peripheral to telemedicine integration but foundational to it.

Kim Y et al. (4) document that limited broadband availability and unstable satellite connectivity are persistent barriers to sustained in-flight telemedicine. Applied inferentially to SSA, this review notes that these barriers are particularly acute on routes over remote or rural airspace which constitute a significant proportion of intra-African flight corridors. The severity of these constraints, however, differs across the region, with better resourced airlines and major international routes more likely to have access to stable connectivity compared with smaller regional carriers including Proflight (Zambia), ASKY Airline (Togo) and Jambojet (Kenya) operating in underserved airspaces. Furthermore, the review found that a number of SSA regional carriers operate some of the oldest fleets globally, with average aircraft ages ranging between approximately 17 and 19 years, significantly higher than global averages which are not compatible with telemedicine adoption (21). For example, South African Airways operates fleets with an average age exceeding 15 years (21). These patterns reflect a broader reliance on second-hand aircraft acquisition and limited fleet modernization across parts of the region. Consequently, these aging aircraft fleets lack the modern avionics and onboard connectivity infrastructure required to support advanced telemedicine systems (19, 21). Nonetheless, this trend is not uniform, as leading carriers such as Ethiopian Airlines have acquired newer fleets, illustrating variation in technological readiness across the region.

This review further established that technological and infrastructural deficiencies intersect with broader economic constraints, limiting telemedicine adoption in the aviation sector. As highlighted in Table 3, the financial barriers extend beyond technological readiness to reflect deeper structural and funding constraints within the aviation market. Studies by (19) and (39) note that high capital costs for onboard telemedicine systems, satellite connectivity and compatible ground-based infrastructure present barriers to implementation. Applied to SSA, these costs are disproportionately prohibitive for smaller regional carriers operating under financial pressure, lacking institutional funding. In contrast to high-income regions where government and industry partnerships subsidize digital health integration (18), SSA airlines often bear full implementation costs. Limited access to capital and reliance on second-hand aircraft further restrict fleet modernization, a prerequisite for effective telemedicine. This finding highlights that aviation telemedicine in SSA represents as much a financing challenge as a technological one.

Additionally, an overlooked dimension of Compatibility in the SSA aviation telemedicine context concerns cultural attitudes toward digital health technologies among both passengers and cabin crew. DOI explicitly incorporates alignment with existing values and past experiences as central to Compatibility assessments, yet the synthesized evidence reveals a near-total absence of research examining how SSA aviation stakeholders perceive and relate to in-flight telemedicine. Broader telemedicine adoption studies within SSA's healthcare systems, including (22, 23) document that variable public trust in digital health platforms, concerns about data privacy and limited prior exposure to technology-mediated care represent culturally grounded Compatibility barriers that have constrained terrestrial telemedicine uptake across the region. Applied inferentially to the aviation context, limited prior exposure to in-flight telemedicine among SSA passengers and crew means that the innovation lacks the experiential anchoring that facilitates perceived Compatibility. Passengers accustomed to limited in-flight health services may not expect or trust telemedicine-mediated care, while cabin crew with no prior experience of operating digital health systems during emergencies may perceive the innovation as culturally and professionally unfamiliar. This absence of accumulated shared experience within SSA's aviation social system is consistent with DOI's contention that innovations with low Compatibility relative to prevailing values and experiences face slower and more contested diffusion trajectories (29). Addressing this dimension requires not only technical and regulatory interventions but deliberate efforts to build institutional familiarity and trust through simulation-based training, stakeholder engagement and transparent communication of telemedicine outcomes within the region's aviation community.

Beyond cultural and organizational barriers, cybersecurity represents a structurally distinct and a consequential threat to aviation telemedicine adoption in SSA that demands policy attention (40). document that in-flight health data transmission across satellite links exposes sensitive biometric and patient records to unauthorized access and system compromise, risks that are acutely heightened in SSA's under-regulated digital environment. Within the DOI framework, these vulnerabilities operate simultaneously across two attributes. They amplify Complexity by introducing technically demanding security requirements that exceed the current capabilities of most SSA carriers, and they undermine Compatibility by creating institutional incompatibility between telemedicine systems and fragmented underdeveloped data protection legislative landscape across SSA member states. Critically, inadequate cybersecurity erodes passenger trust in data confidentiality, complicates regulatory approval processes and generate unresolved cross-border liability exposure when health data is shared across multiple national jurisdictions mid-flight (18). Without explicit cybersecurity governance integrated into regional telemedicine frameworks, the compounding risks will continue to hamper adoption.

Beyond technological and infrastructural barriers, aviation telemedicine as a clinical service model raises additional dimensions that warrant considerations. Quality of care in telemedicine-mediated consultation is contingent on structural clinical workflows that clearly delineate professional responsibilities between cabin crew, ground clinicians and receiving facilities. However, such protocols remain absent across most SSA carriers (11, 15). The absence of defined professional responsibility boundaries raise ethical concerns around accountability, informed consent and standard of care owed to passengers in jurisdictions where telemedicine governance is underdeveloped (18, 38). Reimbursement models represent a further overlooked dimension. Unlike ground-based telemedicine where billing frameworks are increasingly institutionalized, aviation telemedicine lacks established funding mechanisms, leaving airlines to absorb service costs without compensation structures (19, 39). Within DOI, these gaps compound the Complexity and Compatibility challenges already constraining adoption, as potential adopters face only technical uncertainty but unresolved clinical governance, professional liability and financial sustainability challenges that no amount of infrastructure investment alone can resolve.

## 5 Practical implications for policy and practice

The findings of this review carry direct implications for airline operators, aviation regulators and healthcare policy makers across SSA.

### 5.1 Airline operators

- Airline operators across SSA could explore the phased adoption of telemedicine, beginning with the integration of ground-based teleconsultation services, such as those offered by MedAire's Aviation MedLink, into existing flight operations manuals, as these require no upfront onboard hardware investment.
- For carriers already operating satellite-equipped aircraft, such as Ethiopian Airlines and Kenya Airways, formal service agreements with established ground-based medical support providers represent a low-barrier and cost-proportionate entry point toward structured telemedicine adoption.
- Smaller and lower-resource carriers may consider telephone-based medical advisory services as a viable and affordable first step, consistent with communication infrastructure already available across much of the region.
- Adoption could be pursued as a phased and scalable process adjusted to each carrier's fleet profile, route network and financial capacity, rather than modelled on high-investment systems designed for high-income aviation contexts.
- Airlines operators may integrate structured telemedicine protocols into cabin crew training programs, building on current first-aid curricula already mandated by ICAO Annex 6, rather than requiring entirely new frameworks.

### 5.2 Aviation regulators

- Aviation regulators, particularly AFCAC in its coordinating capacity, may consider facilitating a multi-stakeholder consultative process involving national civil aviation authorities, ministries of health and ICAO to develop non-binding model regulations and minimum telemedicine standards for voluntary adoption and domestication within respective national legislative frameworks aligned with the ICAO Global Aviation Safety Plan and related ICAO Aviation Medicine provisions.
- AFCAC could establish minimum technical standards for onboard telemedicine systems, ensuring compatibility across SSA's diverse fleet profiles and route networks, to reduce regulatory fragmentation that currently constrains adoption.
- National civil aviation authorities can work collaboratively with ministries of health to formally recognize aviation as a telemedicine delivery context, enabling coherent cross-sectoral governance of in-flight telemedicine services.
- There is also need for aviation regulators to mandate minimum data protection standards for in-flight health data, drawing on the African Union Convention on Cyber Security and Personal Data Protection (Malabo Convention) as a reference instrument, to safeguard passenger privacy and maintain stakeholder trust in telemedicine systems.

### 5.3 Health policy makers and ministries of health

- Healthcare policy makers and ministries of health may formally recognize aviation as a telemedicine delivery context within national digital health strategies, enabling the inclusion of aviation teleconsultation services within existing public-private digital health funding mechanisms to reduce the financial burden on individual carriers.
- Healthcare policymakers and ministries of health in countries with functional ground telemedicine service can explore formal agreements to extend existing ground-based teleconsultation services to airline operators, leveraging available infrastructure rather than constructing parallel systems.

## 6 Conclusion

The rapid advancement of communication technologies including satellite communications has made network access available anywhere and anytime including in the airspace. This development has enabled in-flight telemedicine to evolve to a sufficiently viable level for both developed and developing contexts. This review has demonstrated that telemedicine holds transformative potential for in-flight emergency care in SSA's aviation sector, and is not only beneficial to patients, but airlines and medical service providers. However, this potential remains largely unrealized and underexplored in scholarly literature. Critically, aviation represents a crucial yet overlooked telemedicine delivery context within SSA's broader digital health landscape. While regional and global health strategies have increasingly prioritized remote and underserved communities, the airspace, traversed daily by thousands of passengers, many carrying undetected or poorly managed chronic conditions, has remained visibly absent from these conversations. This gap is crucial. As the SSA's aviation sector expands rapidly, the absence of structured telemedicine integration means that a growing patient population in transit remains outside the protective reach of the region's evolving digital health infrastructure. Positioning aviation within SSA's digital health agenda is therefore crucial.

Synthesizing evidence across four analytical domains, the review identified six prospects anchored in three DOI attributes (Relative Advantage, Trialability and Observability), collectively signaling that telemedicine is not merely a technological supplement but a structural necessity for a region where aviation business is booming, the burden of chronic diseases is increasing, aviation medical officer shortages are persistent, and ground-based medical support remains unevenly distributed across flight corridors. The challenges documented reveal that adoption barriers in SSA are not simply technical problems awaiting engineering solutions. They demonstrate deeper systemic challenges that demand coordinated policy responses rather than fragmented technological interventions. This review positions SSA's aviation sector at a critical juncture, where the intersection of increasing passenger volumes, a growing burden of chronic diseases, and the expansion of digital health creates both the imperative and the opportunity for the integration of telemedicine solutions. Realizing this potential however demands sustained scholarly attention, harmonized regulatory commitment and targeted investment tailored to the region's specific contextual realities, rather than solutions uncritically derived from high income settings. This review offers several notable contributions. It provides one of the first theoretically grounded syntheses of aviation telemedicine prospects and challenges within SSA's aviation sector. It employs an inferential synthesis methodology that systematically bridges three convergent bodies of adjacent evidence to generate structured insights where direct primary evidence remains limited. Lastly, it applies Rogers' DOI theory within an SSA aviation telemedicine context and generates a stakeholder- differentiated policy framework directly context recommendations at airline operators, aviation regulators and health policymakers respectively.

### 6.1 Areas for future research

The inferential character of this review's evidence base is not a methodological limitation but a diagnostic finding in itself. The paucity of primary empirical research within SSA's aviation health context represents a significant scholarly gap that this review has both exposed and structured. There is a need for primary studies to generate SSA specific in-flight medical emergency epidemiological data, examine passenger and crew perceptions of telemedicine within the SSA aviation context and rigorously evaluate implementation outcomes across the region's diverse aviation markets. Without such evidence, policy and practice will continue to operate on inference borrowed from high-income settings, whose structural conditions bear limited similarities to SSA's realities. This review provides a conceptual and empirical platform upon which that urgent primary research can be built. In conclusion, the study uniquely contributes by laying the groundwork for empirical research, policy development, and implementation in SSA aviation telemedicine. It establishes a basic theoretical, methodological, and policy-oriented framework where none existed before.

### 6.2 Limitations

This review carries several limitations. The absence of primary empirical research within SSA's aviation health context necessitated an inferential synthesis strategy, limiting the directness of evidence. To mitigate this, evidence was drawn systematically across four convergent analytical domains and assessed against a structured transferability protocol to ensure contextual relevance. Restricting inclusion to English-Language sources may have excluded relevant literature from Francophone and Lusophone SSA countries, potentially limiting the geographic and cultural representativeness of the review. This decision was taken to maintain methodological consistency and minimize translation and interpretive bias in the assessment of technical aviation, healthcare and regulatory literature. Future reviews should employ multilingual search strategies and cross-language screening approaches to capture a broader range of regional perspectives. Additionally, the review was not prospectively registered. Findings should therefore be interpreted as a structured evidential foundation for future empirical inquiry rather than definitive conclusions.
