# Supplementary material for: Telemedicine in the sky: prospects and challenges for the aviation sector in Sub Saharan Africa
Source: Front Digit Health. 2026 Jul 23;8:1865607. doi: 10.3389/fdgth.2026.1865607 (PMC13442809; doi:10.3389/fdgth.2026.1865607)
Supplement: Supplementary file 1 [file Table1.docx]

**Supplementary Table 1: Data Extraction Table**

**Total sources: 35**

| **Author (s) / year** | **Geographic settings** | **Study design/ type** | **Population/focus** | **Key findings relevant to this review** | **Prospects/ benefits identified** | **Challenges/ Barriers identified** |
| --- | --- | --- | --- | --- | --- | --- |
| **Domain 1: Global aviation telemedicine studies 16 studies** | | | | | | |
| **Boneli (2017)** | Brazil / Global | Review | Cabin crew; passengers; aviation professionals | Telehealth integration in aviation is a significant advancement in passenger safety; ICT enables video feeds, vital signs and real-time data transmission between crew and ground clinicians | Teleconsultation between crew and ground; multimodal biometric data transmission | Infrastructure and satellite connectivity constraints in aviation settings |
| **Tadayon et al. (2016)** | Canada / Global | Technical study (IEEE Access) | Airlines; telecoms engineers; aviation IT | Cellular network broadband feasible in-flight; speed (4–10 Mbps minimum) and latency are critical constraints affecting telehealth quality | Technical enabler: satellite/cellular connectivity for real-time telehealth | Unstable connectivity; latency constraints critical for real-time telehealth; broadband gaps over remote airspace |
| **Elford (2017)** | Canada / Global | Book chapter | Healthcare teams; cabin crew; ground clinicians | Telehealth improves healthcare team communication; multimodal data transmission improves diagnostic accuracy over verbal communication alone | Remote diagnostic decision support; physician-assisted oversight | Interoperability gaps; communication limitations in remote/constrained environments |
| **Sene et al. (2018)** | France / Global | Empirical study | Cabin crew; aviation medical officers; decision-makers | Decision support systems reduce cabin crew uncertainty and perceived error risk; real-time clinician guidance reduces psychological distress significantly | Clinical decision support; crew psychosocial benefit; reduced fear of medical error | System complexity; digital literacy gaps among cabin crew |
| **Kim & Choi-Kwon (2020)** | South Korea / Global | Empirical study | Ground-based medical support personnel; airline operators | GBMS reduces unnecessary diversions and improves clinical outcomes; financial barriers limit adoption in lower-resource airlines | Ground-based teleconsultation as scalable model for specialist oversight without onboard personnel | High capital costs; lack of institutional funding; coordination gaps between airlines and GBMS providers |
| **Elwezza et al. (2023)** | Global | Conceptual study | Cabin crew; ground medical coordinators; airline operators | Integrated information systems improve medical response and continuity of care; structured telehealth-enabled documentation supports post-event review and medico-legal accountability | Continuity of care; auditable in-flight clinical records; early ground hospital coordination | Interoperability between in-flight and ground systems; fragmented digital infrastructure |
| **Martin-Gill & Doyle (2023)** | USA / Global | Book chapter | Airlines; cabin crew; ground-based medical support personnel; aviation medical officers | Comprehensive account of GBMS as a structured adjunct to in-flight care; GBMS improves clinical decision-making, reduces unnecessary diversions and supports early coordination with receiving hospitals; continuity of care and auditable documentation identified as key operational benefits | Ground-based teleconsultation; continuity of care; early hospital coordination; auditable in-flight clinical records for medico-legal accountability | Availability and coordination gaps between airlines and GBMS providers; limited access to 24/7 physician-staffed ground services; absent physician in majority of cases |
| **MoghadasNian & Manafi (2024)** | Global | Review | Airlines; aviation IT professionals; medical crew | Digital innovations transforming aviation; telemedicine reduces cabin crew distress and perceived error risk; strategic adoption improves operational efficiency | Crew psychosocial benefit; real-time clinical support; brand and operational efficiency gains | Digital literacy gaps; high investment required; regulatory uncertainty |
| **Battineni et al. (2024)** | Italy / Global (top 10 airlines) | Systematic review | Top 10 global airlines; cabin crew; aviation healthcare professionals | Crew training, GBMS and onboard equipment are three pillars of response; telemedicine and real-time teleconsultation represent the future of aviation medicine; documented consultations support medico-legal accountability | Real-time teleconsultation; medico-legal accountability via documentation; brand and trust signaling | Limited crew training; onboard equipment gaps; connectivity requirements for real-time systems |
| **Rajendran & Yasin (2024)** | Malaysia / Global | Conceptual framework | Airlines; passengers; aviation medical officers | FlyHealth integrated platform concept; telehealth improves operational preparedness and brand competitiveness; pre-flight consultations enhance safety | Pre-flight screening; operational preparedness; brand and trust benefits | Financial barriers; regulatory fragmentation; digital health literacy deficits |
| **Bhattacharya & Kaur (2025)** | Global | Book chapter | Passengers; cabin crew; airline operators | Pre-flight telehealth enables early identification of pre-existing conditions; passenger confidence enhanced; evidence cited for 40% faster emergency response vs conventional radio communication | Pre-flight screening; real-time teleconsultation; passenger psychosocial benefit; 40% faster emergency response vs radio | Unclear medico-legal liability; data protection legislation; cybersecurity vulnerabilities; financial barriers |
| **Kim et al. (2025)** | South Korea / Global | Review | Airlines; passengers; voluntary medical professionals; telecoms providers | IMEs occur at ~127/million passengers; physician absent 41.1%; minimum 4–10 Mbps internet speed required; telehealth benefits passengers, airlines and volunteer medical professionals; new business models for insurers and telecoms | Real-time consultation; multimodal biometric data transmission; reduced diversions; passenger confidence; innovative business models | Internet speed and latency requirements; documentation gaps; business model innovation needed |
| **Shafik (2025)** | Global | Book chapter | Airlines (Emirates, KLM, Lufthansa); passengers; cabin crew; ground clinicians | Emirates and KLM have deployed telemedicine on long-haul fleets, resolving emergencies without diversion; Lufthansa pre-flight consultations enable individualised care plans; operational efficiency and safety enhanced | Real-time teleconsultation; pre-flight screening; individualised care plans; reduced diversions; operational and safety gains | Satellite connectivity costs; regulatory compliance; equitable access across airline types |
| **Dedouit et al. (2007)** | France / Global | Review | Physician passengers; French legal system; airlines | Legal obligations for physician passengers depend on jurisdiction; telehealth-enabled documentation supports post-event review and medico-legal accountability | Auditable in-flight clinical records for post-event review; medico-legal accountability via documentation | Medico-legal ambiguity; jurisdictional inconsistency across borders |
| **de Caprariis & Di Maio (2021)** | USA / Global | Review | Physicians; airlines; aviation legal practitioners | Medico-legal liability for emergency care on aircraft remains unclear internationally; absence of harmonised cross-border telehealth frameworks impedes integration; documented consultations improve accountability | Documented telehealth consultations as medico-legal safeguard | Unclear medico-legal liability; absence of harmonised cross-border telehealth regulatory frameworks |
| **Eleimat & Oszi (2025)** | Global / Jordan & Hungary | Review | Aviation IT systems; airlines; regulators | Cybersecurity critical for aviation including health data transmitted via onboard systems; fragmented policy limits regulatory standardisation; data breach risk undermines stakeholder trust | Addresses enabling condition for safe telehealth: robust cyber governance and data protection | Weak cybersecurity; inadequate data protection legislation; fragmented inter-agency coordination between aviation and health authorities |
| **Domain 2: Global Aviation Medicine- In flight medical emergencies and clinical management (11 sources)** | | | | | | |
| **Ferrer-Roca et al. (2002)** | Spain / Global | Conceptual review | Airlines; aviation medical officers; passengers | Medical incidents ~10–50/million passengers; onboard kit limited to 3 items; telemedicine presents opportunity for remote diagnostic support | Real-time ground consultation; transmission of patient data to clinicians | Limited broadband; unclear cross-border regulatory framework; medico-legal liability ambiguity |
| **Martin-Gill et al. (2018)** | USA / Global | Narrative review | Airlines; cabin crew; volunteer medical professionals; passengers | Comprehensive review of IME epidemiology, management and outcomes; syncope and cardiovascular events predominate; GBMS improves decision-making and reduces diversions | Ground-based teleconsultation as key adjunct to in-flight care | Limited onboard resources; absent physician in majority of cases; variable airline preparedness |
| **Bhagavath & Al Shukaili (2023)** | Oman / Global | Retrospective cohort | Commercial airline passengers; cabin crew; airline medical officers | Commercial aviation growth increases IME incidence; outcomes depend on crew training, equipment and access to GBMS; highlights growing demand for telehealth solutions | Establishes epidemiological basis and demand for telehealth in aviation | Limited onboard resources; absent physician; variability in airline preparedness |
| **Lo et al. (2021)** | Taiwan | Retrospective cohort | In-flight and ground-based emergency patients at Taoyuan Airport | In-flight emergencies differ from ground-based in acuity and clinical demand; early ground coordination improves post-landing patient outcomes | Continuity of care; early hospital coordination supported by telehealth | Interoperability gaps; fragmented digital infrastructure between in-flight and ground systems |
| **do Nascimento et al. (2021)** | Global (multi-country) | Systematic review & meta-analysis | Airline passengers across the globe | IME incidence ~0.44/million passengers; physician absent in 41.1% of cases; syncope, respiratory, and GI events most common; aircraft diversion rare but operationally costly | Quantifies scale of problem necessitating telehealth; supports case for GBMS and telemedicine integration | No mandatory international reporting system; highly variable data across airlines and jurisdictions |
| **Ceyhan & Mensekse (2021)** | Turkey / Global | Review | Commercial airline passengers; cabin crew | Respiratory distress, syncope, GI complaints and cardiovascular events most frequently reported; management constrained by limited resources and absent physicians | Clinical demand justifying telehealth deployment; need for remote diagnostic support | Limited onboard resources; absent physician in most flights; environmental physiological constraints at altitude |
| **Hinkelbein et al. (2021)** | Europe | Survey study | European airlines; cabin crew; aviation medical officers | Significant variability in onboard medical equipment across European airlines; physician absent in most flights; cabin crew training inadequate for complex emergencies | Highlights critical need for telehealth to supplement limited onboard equipment and training | Inadequate cabin crew training; shortage of aviation medical officers; onboard equipment gaps |
| **Pavlovic (2023)** | Serbia / Global | Review | Cabin crew; volunteer medical professionals; passengers | Physician absent in most commercial flights; cabin crew training critical but often inadequate; psychological distress among crew managing emergencies without specialist support | Crew psychosocial benefit from telehealth support; physician-assisted oversight via GBMS | Cabin crew training inadequacy; shortage of aviation medical officers; psychological burden on crew |
| **Aguiar et al. (2025)** | Global | Review | Passengers with allergic conditions; cabin crew; airline medical officers | In-flight allergic emergencies growing with rising passenger volumes, aging demographics and chronic disease burden; management complicated by limited onboard resources | Pre-flight screening of at-risk passengers; telehealth to support management of allergic emergencies in-flight | Limited onboard resources for allergic emergencies; absent specialist; inadequate crew training for allergy management |
| **Alves et al. (2025)** | Global (84 airlines, 6 continents) | Cohort study | 77,790 in-flight medical events across 84 airlines; 3.1 billion passenger boardings (2022-2023) | Largest IME study ever conducted; physician absent in 41.1% of cases; significant variation in IME rates (7-257/million passengers); GBMS critical for decision-making; onboard AED and crew training important | GBMS and telehealth as essential complement to onboard care; highlights variation in airline preparedness | Variable airline preparedness; limited equipment; not all airlines partner with GBMS; regulatory gaps in medical kit requirements |
| **Soh et al. (2025)** | Singapore / Global | Review | Aviation medical officers; airlines; regulatory bodies | IME rate projected at 10-50/million passengers and rising; aviation medicine critical for safeguarding safety; telemedicine integration key future direction; regulatory and training frameworks must evolve | Aviation medicine and telehealth as integrated safety framework; pre-flight and in-flight risk management | Regulatory evolution needed; training gaps; limited specialist workforce in aviation medicine |
| **Domain 3: Aviation medicine in developing of resource constrained contexts- Sub Saharan African (5 sources)** | | | | | | |
| **Njoroge & Samunderu (2021)** | Sub-Saharan Africa | Book chapter | African airlines; aviation regulators; policymakers | SSA aviation faces regulatory fragmentation, limited GBMS access, persistent connectivity gaps; shortage of aviation medical officers; financial constraints restrict fleet modernisation | SSA context establishing need for telehealth to address shortage of GBMS and aviation medical officers | Regulatory fragmentation; financial barriers; connectivity gaps along intra-African corridors; shortage of aviation medical officers |
| **Parker & Mahomed (2019)** | South Africa | Retrospective cohort | Passengers of South African national airline | Disease burden of in-flight events on African carrier reflects SSA passenger population; context-specific screening superior to universal protocols derived from high-income settings | Establishes SSA-specific epidemiological basis for pre-flight screening and risk stratification | SSA-specific disease burden (NCD and communicable diseases); limited local data; gaps in context-specific protocols |
| **Wembonyama et al. (2021)** | DRC / Global | Narrative review | SSA passengers with sickle cell disease; haematology clinicians | Elevated risk of in-flight complications for SSA passengers with sickle cell disease; pre-flight risk stratification essential and tailored to SSA disease profile | Pre-flight screening and tailored risk stratification for SSA-specific conditions (sickle cell, NCD) | Inadequate pre-flight screening protocols; limited awareness of SSA disease burden in aviation medicine |
| **Taiwo et al. (2024)** | West & North Africa | Case study | Airport emergency response personnel; aviation operators | Human factors critical determinants of aviation emergency outcomes; training deficits and limited aviation medical officer availability prevalent in West and North African airports | Operational need for telehealth to compensate human factor limitations in African aviation contexts | Inadequate crew training; shortage of aviation medical officers; limited emergency infrastructure at airports |
| **Mikula et al. (2025)** | Sub-Saharan Africa | Conference paper (NTAD 2025) | African airlines; aviation industry stakeholders | SSA carriers operate some of the oldest fleets globally (avg 17-19 years); significant heterogeneity: Ethiopian Airlines leads vs smaller regional carriers; regulatory fragmentation across AFCAC member states constrains harmonisation | Heterogeneous adoption readiness — leading carriers (Ethiopian Airlines, Kenya Airways) characterised as potential early adopters for telehealth | Ageing fleet (avg 17-19 years); incompatible onboard systems; regulatory fragmentation; limited fleet modernisation capacity |
| **Domain 4: Telemedicine adoption in Sub Saharan Africa’s broader healthcare systems (3 sources)** | | | | | | |
| **Dodoo et al. (2022)** | Sub-Saharan Africa | Review (66 studies) | SSA health systems; patients; health workers; policymakers | Unbalanced telemedicine progress across SSA; technological, organisational, legal-regulatory, financial and cultural barriers identified; perceived clinical utility key adoption determinant | SSA telemedicine adoption patterns transferable to aviation telehealth adoption; perceived utility drives adoption | Technological barriers; legal-regulatory gaps; financial constraints; inadequate funding; digital infrastructure deficits; cultural factors |
| **Chilunjika et al. (2024)** | Zimbabwe (SSA) | Empirical study | Zimbabwe public health system; health workers; patients | Telemedicine improves access to care, clinical outcomes and timeliness of interventions in resource-constrained SSA settings; perceived clinical utility drives adoption | SSA transferable evidence: telehealth clinical utility in resource-constrained settings supports aviation application | Resource constraints; infrastructure deficits; digital literacy gaps; implementation barriers of digital health applications |
| **Benecke et al. (2026)** | Africa | Review | African health systems; digital health innovators; policymakers | AI, telemedicine and personalised medicine improving healthcare delivery across Africa; digital health transformative for resource-constrained settings; supports case for expansion to aviation | SSA transferable evidence: telehealth improves healthcare delivery and access in Africa broadly | Regulatory gaps; financing constraints; infrastructure deficits; need for equity-focused implementation |

***Abbreviations:*** *SSA = Sub-Saharan Africa; GBMS = Ground-Based Medical Support; IME = In-flight Medical Emergency; AFCAC = African Civil Aviation Commission; DOI = Diffusion of Innovations; NCD = Non-communicable Disease; ICT = Information and Communication Technology; LMIC = Low- and Middle-Income Country; DRC = Democratic Republic of Congo; AED = Automated External Defibrillator.*
